# COVID-19 Post-Exposure Evaluation (COPE) Study: Assessing the Role of Socio-Economic Factors in Household SARS-CoV-2 Transmission within Campania Region (Southern Italy)

**DOI:** 10.3390/ijerph191610262

**Published:** 2022-08-18

**Authors:** Ivan Gentile, Martina Iorio, Emanuela Zappulo, Riccardo Scotto, Alberto Enrico Maraolo, Antonio Riccardo Buonomo, Biagio Pinchera, Giuseppina Muto, Carmela Iervolino, Riccardo Villari, Nicola Schiano Moriello, Maria Michela Scirocco, Maria Triassi, Mariano Paternoster, Vincenzo Russo, Giulio Viceconte, Federico II COVID-Team

**Affiliations:** 1 Department of Clinical Medicine and Surgery, University of Naples Federico II, 80138 Naples, Italy; 2 Department of Economist, Roma Tre University, 00154 Rome, Italy; 3 First Division of Infectious Diseases, Cotugno Hospital, AORN dei Colli, 80131 Naples, Italy; 4 Ninth Division of Infectious Diseases, Cotugno Hospital, AORN dei Colli, 80131 Naples, Italy; 5 Department of Public Health, University of Naples Federico II, 80138 Naples, Italy; 6 Department of Advanced Biomedical Sciences, University of Naples Federico II, 80131 Naples, Italy; 7 Department of Medical Translational Sciences, University of Campania “Luigi Vanvitelli”, Monaldi Hospital, 80131 Naples, Italy

**Keywords:** COVID-19, SARS-CoV-2, household, poverty, regional studies, Campania (Italy)

## Abstract

Campania is the sixth poorest region of Italy, and it is the region with the highest income inequality. The secondary attack rates of SARS-CoV-2 among households are found to be substantially heterogeneous among published studies and are influenced by socio-economic factors. We conducted a retrospective study to describe the role of socio-economic factors in the household transmission of SARS-CoV-2 among patients living in Campania Region and referring to “Federico II” Hospital. We interviewed 413 subjects followed-up for COVID-19 between the 8 March 2020 and the 24 May 2021 with the aim to collect demographic, clinical, economic, and social data regarding their household and the index cases. The variables associated with SARS-CoV-2 attack rate higher than 50% among households were higher age (*p* = 0.023) and higher Charlson Comorbidity Index of the index case (*p* = 0.023) and, for household characteristics, higher number of families per house (*p* = 0.02), location of the houses in Naples’ suburbs (Chi^2^ = 5.3, *p* = 0.02) and in Caserta City area (Chi^2^ = 4, *p* = 0.04), and renting the house compared to owning it (Chi^2^ = 5.83, *p* = 0.01). This study confirms the finding described by other authors that household transmission of SARS-CoV-2 is correlated with the income inequality of the analyzed geographical area as well as with the indicators of health and economic wealth of the families, and this correlation also applies to the Campania Region.

## 1. Introduction

Campania is one of the 20 regions of Italy, and its territory is divided into five provinces, namely Naples, Caserta, Avellino, Benevento, and Salerno, by the name of the main city in which the government of each province is held. The Metropolitan City of Naples consist of Naples’ Municipality (the county seat) and its suburb (other municipalities administered by the Province of Naples). The Municipality of Naples is divided in 10 health districts (*Distretti Sanitari*) from n.24 to n.33, which are under the jurisdiction of the Local Health Authorities (ASL).

In the city of Naples, the welfare basin of the ASL includes more than 900,000 citizens distributed over an area with high population density. Citizens show an old age index of 150%, the average age of the population in some districts exceeds the Italian average by twice, and the number of people with clinical and socio-economic frailty (e.g., chronic diseases, pregnancy, rare diseases, prisoners) reaches 600,000 individuals out of the total population in accordance with ASL data, updated on 1 January 2019 [1,2].

At the time of this study, and precisely at the date of 31 March 2021, the cumulative number of positive cases of SARS-CoV-2 virus within the welfare basin of the ASL of Naples City since the beginning of the pandemic was 57,822 compared to 49,232 recovered. On that date, 304 people were hospitalized in ordinary hospitalization, 26 in intensive care, 5213 in home isolation, and 2095 were assisted at home by health services [1].

These data show that, as of the date of the survey, approximately 85% of the virus-positive patients were at home either because they were in isolation or being cared by public health services at home. This figure is relevant for the present discussion since the home is by its nature a favorable place for contagion within small groups of related persons.

Secondary attack rate (SAR) is defined as the incidence of an infection among susceptible people within a specific group, and according to international literature [3], the SARs are substantially heterogeneous among households worldwide.

According to a 2020 meta-analysis by Lei H. and colleagues [4], the secondary attack rates (SAR) of SARS-CoV-2 among households were substantially heterogeneous among published studies, ranging from 4.6% to 90.0% (I2 = 96%), with pooled rate of 27% (95% CI: 21–32%) [3]. However, according with recent international studies, COVID-19 transmission and outcomes may be correlated not only with health factors but also with socio-economic variables, such as population density, household size, social distress, or Gini inequality index [5,6], among others.

According to the abovementioned insights from literature, and given a brief outlook of the area of interest throughout this section, the present study is aimed at observing the relationship between socio-economic factors and SARS-CoV-2 transmission within households in the Campania Region (Southern Italy).

### Socio-Economic Background

**Figure 1:**
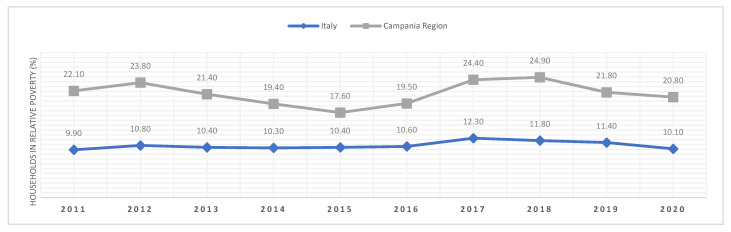
Incidence of household-relative poverty. Source: Authors’ elaboration from ISTAT (data extracted on 25 May 2022 12:05 UTC (GMT)).

Campania is the sixth poorest region of Italy in terms of gross domestic product (GDP) per capita, the third in terms of household consumption, and its family poverty rate is about twice the national rate (Figure 1). The Region is overpopulated and suffers a high unemployment rate and poor social services and infrastructure in addition to low institutional quality and a wide presence of criminal organizations [7,8]. The metropolitan city of Naples is the second-largest populated city in Italy and one of the largest in Europe, with an estimated population of over 4 million inhabitants and a population density that reaches in some areas the density of the big Asian cities [9]. Campania has also a higher Gini score among the 20 Italian regions, according to the Italian National Institute for Statistics (ISTAT): this means that Campania is the region with the highest wealth inequality in Italy [10].

In this context, life expectancy in Campania is the lowest in Italy [7,8]. The overall number of deaths for all causes in 2021 was the third highest in Italy, according to ISTAT [8]. However, since the outbreak of SARS-CoV-2 pandemic in 2020, Campania is, to date, the third Italian region for number of cases (1.7 millions) but the last for mortality rate (0.63), according to Center for Systems Science and Engineering (CSSE) at Johns Hopkins University [11]. Moreover, during the COVID-19 pandemic, a significant reduction rate in all the clinical activities, including both non-invasive tests and cardiac invasive procedures, has been shown in the Campania Region [12,13,14].

At present, there are no studies on the evaluation of the household transmission of SARS-CoV-2 in the healthcare basin of Campania nor data available in the literature that correlate the risk of transmission of the infection with the socio-economic variables of the population of Campania.

## 2. Patients and Methods

### 2.1. Study Aims

We conducted a retrospective study to describe the interactions between socio-economic factors and household transmission of SARS-CoV-2 among patients living in the Campania Region and referring to “Federico II” University Hospital, our institution.

To reach our aim, we collected clinical, demographic, and economic variables, and we correlated them to the SARS-CoV-2 attack rate (AR) among the observed households, assuming that SARS-CoV-2 household AR is defined as the number of new cases of SARS-CoV-2 infection in each household among the number of subjects living within the same household in the time interval of two weeks.

### 2.2. Study Setting and Patients’ Selection

The present study was conducted at “Federico II” University Hospital sited in Naples (Campania), which is the largest University Hospital in Southern Italy and a referral hospital for COVID-19 in Campania. It is the only center for COVID-19 in pregnancy in the Region and has a catchment area of over 2 million people that inhabit the city of Naples and its metropolitan area.

We screened electronic records from outpatients and inpatients that were followed-up at our institution for COVID-19 between 8 March 2020 and 24 May 2021, by using ICD-9 codes for SARS-CoV-2 infection, COVID-19 pneumonia, and COVID-19-related ARDS as filters for the diagnosis-related group (DRG) list.

### 2.3. Sample Size Calculation

For the calculation of the sample size, it was considered that the number of subjects with SARS-CoV-2 infection residing in the area of competence of the Naples’ Health Authority assisted at home as of 31 March 2021 is approximately 2000, as reported by the epidemiological surveillance bulletins [1,2]. For each positive subject, a number of family/domestic contacts of 3 subjects was estimated: the starting population was therefore estimated at around 6000 subjects. Considering a SAR between family/domestic contacts estimated from studies available in the literature to be around 21% [4], a confidence interval of 5% was calculated, multiplying a Z-score of 1.96 by the ratio between the standard deviation of the population and the square root of the size of the population. A sample number of subjects out of a population of 400 subjects was then calculated by setting the confidence interval at 4% with a confidence level of 95%. Considering a drop-out/attrition rate of at least 20%, the total number of subjects to be included in the study was estimated around 400 subjects.

### 2.4. Definitions

The definitions used to pinpoint the main variables are listed below:

*Index case*: the first member of the household to report COVID-19 symptoms;

*Household*: a house and its occupants regarded as a unit;

*Head of the household (HOH)*: the person in the household with the highest income;

*Disabled individual*: a person that has a legally recognized disability according to Italian law;

*Self-isolation*: isolation from the rest of the household as prescribed by the Italian Ministry of Health (single room with a dedicated bathroom in the same home or in another home);

*Hospital admission*: admission to hospital within the period of SARS-CoV-2 infection, from the first known diagnosis to the first negative test;

*Death*: death attributed to COVID-19

*Charlson Comorbidity Index*: a score to predict 10-year survival in patients with multiple comorbidities, created by M.E. Charlson and colleagues [15].

### 2.5. Data Collection

Subjects were contacted by telephone. Up to five attempts in five different days were performed for subjects that did not answer at the first contact. If a subject was not reachable after five attempts, it was considered as not reachable and therefore excluded from the study. Subjects were asked if they were able to identify an index case of their household and if they were a case index themselves.

The following data about index cases were then collected and were set as study variables: age; sex; pregnancy status; day of the first test for SARS-CoV-2; day of symptoms onset; vaccination status; home isolation; hospital admission; death; disability; and comorbidities for Charlson Comorbidity Index calculation [15]. Moreover, the following data about the household were collected: dimension in square feet; number of rooms; number of bathrooms; number of disabled people; number of families living together; type of house property (owned, on rent); and household location (City of Naples and with health district; Naples’ suburbs; City and province of Caserta, Avellino, Benevento, or Salerno). Subjects were also asked to report education level, employment, and annual income of the HOH. Such variables were selected among the 129 indicators of equal and sustainable wealth provided by the Italian National Institute for Statistics (ISTAT) and were chosen based on the facility of their collection through telephonic interview and their potential relation to COVID-19 transmission.

In order to standardize and generalize the results, the economic variables were referred to the HOH since ISTAT’s surveys use the HOH’s characteristics as a proxy of the household wealth.

### 2.6. Statistical Analysis

Continuous variables were described as: number of subjects (n), mean, standard deviation (SD), median, and interquartile range (IQR). Categorical variables were described as counts and percentages.

Categorical variables were confronted using chi-squares test and Fisher’s exact test when appropriate; continuous variables were confronted to continuous variables using Mann–Whitney U test. A significance level of 0.05 was set for the interpretation of the results. Statistical analyses were performed using IBM SPSS Statistics version 27 (SPSS Inc., Chicago, IL, USA).

### 2.7. Ethical Considerations

The study was conducted according to 1964 Declaration of Helsinki and its later amendments. The study was approved by the Ethical Committee of the University of Naples “Federico II” (protocol n. 180/21).

## 3. Results

We contacted 413 subjects diagnosed as COVID-19-positive between 8 March 2020 and 24 May 2021, of which 141 had been hospitalized for COVID-related complications at our institution, and the rest of them had been followed-up as outpatients; 161 (39%) of the subjects were not reachable, of which 17 had died; 21 of the interviewed patients (5%) refused to be interviewed. A total of 215 subjects were interviewed from 215 different households. A total of 195 of them (90%) were able to identify the index case, and in 146 cases (68%), interviewed subjects identified themselves as index cases.

The median age of index cases was 49 years (IQR 34–62). In 109 cases (50.7%), the index was identified as the HOH, while in 97 cases (45%), the index case corresponded to another member of the household, and in 9 cases (4%), interviewed subject could not identify any HOH. Out of 195 identifiable index cases, 23 (11%) were pregnant; 10 (5.1%) had disability; 157 (80%) did not receive any SARS-CoV-2 vaccine doses, 27 (13.8%) received 1 dose, and 7 (3.6%) two doses; 143 (73.3%) managed to provide self-isolation at home; 125 (64%) were hospitalized for any reason. Median dimension of the households from which index cases were identifiable was 100 square feet (IQR 80–120), with a median of three rooms (IQR 2–4) and two bathrooms (IQR 1–2). Among 195 index cases, 95% had Italian nationality, 65% were homeowners, and 27.2% on rent. Regarding HOHs, 66 (36.3%) had a high school diploma, 50 (27.5%) a middle school diploma, 52 (28%) had a university degree, and 14 (7.7%) reported only elementary education. The largest part of the HOHs had a permanent job (30.4%), and 12% were unemployed, with the largest part of them (41%) reporting an annual income lower than EUR 15,000.

Among the 195 index cases, a median of two people living in each household (IQR 1–3) and a median of one person diagnosed with COVID-19 (IQR 0–2) were reported by the interviewed subject, with a total of 463 subjects living with the identified index cases; 235 of them were diagnosed with COVID-19, with an overall AR of SARS-CoV-2 infection of 50% among household contacts of the index cases.

According to our statistical analysis, the variables that resulted as significantly associated with a SARS-CoV-2 AR higher than 50% among household members were higher age (*p* = 0.023) and higher Charlson Comorbidity Index of the index case (*p* = 0.023) and, for household characteristics, higher number of families per house (*p* = 0.02), location of the houses in Naples’ suburbs (Chi^2^ = 5.3, *p* = 0.02) and in the Caserta City area (Chi^2^ = 4, *p* = 0.04) compared to Naples City and other cities of the Campania Region, and renting the house compared to owning it (Chi^2^ = 5.83, *p* = 0.01).

No significant correlations were found between AR and structural characteristics of the houses and socio-economic level of the households in term of employment, educational level, and annual income of the HOH. The results are displayed in Table 1.

## 4. Discussion

As we know from previous large studies conducted on a global scale, several socio-economic variables are significantly correlated with COVID-19 diffusion and outcome. As an example, in their analysis, Breitling and colleagues described the correlation between the weekly reproductive ratio of SARS-CoV-2 across 170 countries worldwide and extreme poverty, average household size, and Gini coefficient [16]. In fact, identifying the contexts, such as the family or more precisely the household, in which potential “supercontagious” events can be recognized and analyzing them can give an indication of how crucial social interactions are in influencing the risk of transmission [3].

Shifting from the global to the national scale, and keeping focused on socio-economic variables, in a Brazilian study of 2020, for example, incidence and mortality of COVID-19 were found to be positively correlated with population density and with the coefficient of income and income inequality (Gini coefficient) across Brazilian federative states [17].

As in the case of Brazil, Kumar et al. (2020) selected Indian states as the base unit for their research [5]. They used the principal component analysis (PCA) to reveal the main socio-economic factors correlated with the contagion. They found out that the variables with the strongest correlation with the transmission of COVID-19 are not only population density but also the contribution of the state to the country gross domestic product (GDP). In this case, urbanization and industrialization *per se* represent a favorable element for infection not so much linked to direct contacts within small groups, mostly family members, but linked to participation in economic activities even during peaks of infection.

Remaining within national contexts, a U.S. analysis conducted at district level showed that the incidence of the virus and associated deaths are positively correlated with certain indicators typical of “distressed communities”. Specifically, it was shown that there is a negative correlation with education and a positive correlation with the presence of African Americans [6].

In our analysis, we found that the transmission of COVID-19 among households is influenced by higher age (*p* = 0.023) and Comorbidity Index of index cases (*p* = 0.023). This can be related to the higher requirement of health assistance during COVID-19 that affects the effectiveness of isolation from the rest of the family.

As for the economic variables, we could not find any significant correlation between SARS-CoV-2 AR and the annual income of the families. Nonetheless, we noticed that the annual income is often under-reported in epidemiological studies since subjects often have undeclared sources of income. Moreover, many subjects interviewed in our studies refused to give such personal information. Thus, we included other indirect variables of economic wealth to our analysis, such as house dimension, educational level, type of house possession, number of families living in the same household, and house location. The higher prevalence of COVID-19 we found in households composed by more than a single family (*p* = 0.02) can be explained with the fact that this is both an indicator for house overcrowding and of poverty since extended families are an old and very widespread method used to mitigate poverty in several areas [18]. Similarly, renting the house instead of owing it can be seen as a proxy of lower socioeconomic status and has been already identified as an indicator of poor health status by other studies [19].

As for correlation with location of the household in Naples’ suburbs or in Caserta city area and the increased prevalence of COVID-19 infection in the households included in the study, we must consider that the Campania Region has the higher Gini score among the 20 Italian regions, as stated above [10]. This means that Campania is the region with the highest income inequality in Italy, with the areas of Naples’ suburbs and Caserta reaching the lowest rankings in Italy for wealth indicators, such as life expectancy, educational level of the population, and employment, according to ISTAT [20]. In light of that, it is not surprising to find a higher prevalence of COVID-19 infections among households located in these areas, in line with the findings of Breitling and Demenech [16,17]. In fact, although our findings cannot directly correlate SARS-CoV-2 prevalence with direct economic variables (e.g., Gini coefficient, annual income), we indirectly reached similar conclusions by considering the economic background of the geographic areas already described by previous statistic reports.

We could not find any correlation between household transmission and socio-economic level of the HOH (annual income, level of education, and employment). We think that this could be related to a reporting bias from interviewed subjects due to difficulty to understand the definition of HOH and also to the misreport of some indicators since undeclared source of income and irregular jobs are very frequent in the analyzed areas.

As described above, our study is limited by its the retrospective nature and the data collection process (i.e., interviews), which might have led to several instances of reporting bias since the precise estimation of AR was hindered by the fact that the identification of the index case and the secondary cases are reported by the interviewed subjects and not verified by the investigators. According to that, it was impossible to estimate the SAR, which is the outcome that is commonly used in household transmission studies.

Although limited by the methodology (retrospective nature, data collection with patient interview, impossibility to calculate SAR) and the high risk of reporting bias, this is the first study to describe the role of socio-economic factors for COVID-19 transmission in the Campania Region.

## 5. Conclusions

Our study confirms that household transmission of SARS-CoV-2 is correlated with economic inequality of the analyzed geographical area as well as with the indicators of health and economic wealth of the families. The age and the number of comorbidities of the index cases as well as the number of families living in the same house, renting the house despite owning it, and living in specific geographical areas of the Campania Region are associated with a higher prevalence of SARS-CoV-2 infection.

These findings should be taken into account not just by healthcare professionals but mainly by the policy makers not only in the healthcare field; in order to reduce SARS-CoV-2 transmission and the spreading of future airborne transmitted infectious diseases, they should improve assistance to elderly and to frail patients and also implement policies and measures to reduce economic inequality in analyzed areas.

The results confirm the importance of continuing to investigate socio-economic factors that have the potential to drive the spread of the contagion and, at the same time, suggest the need to continue working to improve investigation tools and methodologies in this direction.

## Figures and Tables

**Table 1:** Study results.

	*Index Cases* *N = 195*	*AR* *<0.5*	*AR* *≥0.5*	*Chi-Square/Fisher’s Exact Test (df = 1)*	*Significance*
***Females*** *, n (%)*	94 (51.4)	43 45.7)	51 (54.3)	1.4	*p* = 0.1
***Pregnant*** *, n (%)*	23 (11.8)	13 (60)	9 (40)	3.21	*p* = 0.2
***Disabled*** *, n (%)*	10 (5.1)	1 (12.5)	7 (87.5)	3	*p* = 0.2
***SARS-CoV-2 vaccination*** *, n (%)*				3.83	*p* = 0.1
*Vaccination*	31 (17)	8 (25.8)	23 (74.2)		
*No vaccination*	150 (82)	67 (44.7)	83 (55.3)		
***Self-isolation*** *, n (%)*	143 (73.3)	53 (40)	80 (60)	0.68	*p* = 0.7
***Hospital admission*** *, n (%)*	125 (64.1)	51 (42)	70 (53)	0.73	*p* = 0.7
***Deaths*** *, n (%)*	0 (0)	0 (0)	0 (0)	1	*p* = 1
***Age*** *, years, median (IQR)*	49 (34–62)	39 (29-61)	52 (35–62)		*p* = 0.02
***Charlson Index*** *, median (IQR)*	0 (0–2)	0 (0–2)	1 (1)		*p* = 0.02
***House dimension*** *, square feet, median (IQR)*	100 (80–120)	100 (80–130)	100 (80–110)		*p* = 0.1
***Number of rooms*** *, median (IQR)*	3 (2–4)	3 (2–4)	3 (2.25–4)		*p* = 0.1
***Number of bathrooms*** *, median (IQR)*	2 (1–2)	2 (1–2)	2 (1–2)		*p* = 0.6
***Number of disabled inhabitants*** *, median (IQR)*	0 (0–1)	1 (0–1)	1 (0–1)		*p* = 0.4
***Number of families per household*** *, median (IQR)*	1 (1)	1 (1–2)	1 (1)		*p* = 0.02
***House property***				5.83	*p* = 0.01
*Owned, n (%)*	128 (65.5)	60 (35)	63 (36)		
*On rent, n (%)*	53 (27.2)	14 (8)	35 (20)		
***House position***					
*City of Naples*	20 (26)	7 (37)	13 (63)	0.6	*p* = 0.5
• *District 24—Chiaia, Posillipo, S. Ferdinando, isola di Capri*	3 (1.5)	2 (7.1)	1 (3.6)	0.6,	*p* = 0.4
• *District 25—Bagnoli, Fuorigrotta*	3 (1.5)	0 (0)	3 (10.7)	3	*p* = 0.1
• *District 26—Pianura, Soccavo*	5 (2.6)	2 (7.1)	3 (10.7)	0.1	*p* = 0.6
• *District 27—Arenella Vomero*	6 (3.1)	3 (10.7)	2 (7.1)	0.45	*p* = 0.4
• *District 28—Chiaiano, Piscinola, Marianella, Scampia*	2 (1)	1 (3.6)	0 (0)	1.2	*p* = 0.5
• *District 29—Colli Aminei, San Carlo all’Arena, Stella*	1 (0.5)	0 (0)	1 (3.6)	0.9	*p* = 0.5
• *District 30—Miano, Secondigliano, S. Pietro a Patierno*	3 (1.5)	2 (7.1)	1 (3.6)	0.5	*p* = 0.4
• *District 31—Avvocata, Montecalvario, Pendino, Mercato, Porto*	2 (1)	1 (3.6)	1 (3.6)	0.01	*p* = 0.7
• *District 32—Barra, S. Giovanni, Ponticelli, Insediamento 167*	5 (2.6)	2 (7.1)	3 (10.7)	0.1	*p* = 0.6
• *District 33—Vicaria, S. Lorenzo, Poggioreale*	0 (0)	0 (0)	0 (0)		
*Naples’ suburbs*	140 (71)	52 (37)	88 (63)	5.3	*p* = 0.02
*Caserta area*	21 (11)	13 (62)	8 (38)	4	*p* = 0.04
*Benevento area*	6 (3.4)	3 (50)	3 (50)	0.18	*p* = 0.5
*Salerno area*	8 (4.5)	5 (62.5)	3 (37.5)	1.5	*p* = 0.2
***Level of education of the HOH***					
*Elementary school, n (%)*	14 (7.7)	9 (64.3)	5 (35)	3	*p* = 0.8
*Middle school, n (%)*	50 (27.5)	21 (42)	29 (58)	0.1	*p* = 0.52
*High school, n (%)*	66 (36.3)	27 (43)	36 (57)	0.002	*p* = 0.5
*University degree, n (%)*	52 (28)	18 (36.7)	31 (63.3)	0.9	*p* = 0.2
***Employment of the HOH***					
*Permanent job, n (%)*	57 (32)	20 (36.4)	35 (63.6)	1.25	*p* = 0.2
*Temporary work, n (%)*	17 (9.6)	8 (50)	8 (50)	0.4	*p* = 0.3
*Self-employment, n (%)*	36 (20)	17 (47.2)	19 (52.8)	0.5	*p* = 0.3
*Unemployed, n (%)*	23 (13)	12 (52.2)	11 (47.8)	1	*p* = 0.2
*Retired, n (%)*	41 (23)	13 (33.3)	26 (66.7)	1.6	*p* = 0.1
***Annual income of the HOH, EUR***					
*<15.000, n (%)*	48 (41)	20 (47.7)	28 (58.3)	0.13	*p* = 0.4
*15.001–28.000, n (%)*	41 (35)	15 (40.5)	22 (59.5)	0.2	*p* = 0.4
*28.001–55.000, n (%)*	17 (15)	9 (52.9)	8 (47.1)	0.7	*p* = 0.3
*55.001–75.000, n (%)*	5 (4.3)	4 (80)	1 (20)	2.8	*p* = 0.1
*>75.000, n (%)*	4 (3.5)	0 (0)	3 (100)	2.4	*p* = 0.2

AR, attack rate (the number of new cases of SARS-CoV-2 infection in each household divided for the number of subject living within the same household in the time interval of two weeks); HOH, head of the household; IQR, interquartile range.

## Data Availability

The data presented in this study are available on request from the corresponding author. The data are not publicly available due to privacy issues.
